# Patterns of entropy production in dissolving natural porous media with flowing fluid

**DOI:** 10.1371/journal.pone.0204165

**Published:** 2018-09-20

**Authors:** Y. Yang, S. Bruns, S. L. S. Stipp, H. O. Sørensen

**Affiliations:** Nano-Science Center, Department of Chemistry, University of Copenhagen, Copenhagen, Denmark; VIT University, INDIA

## Abstract

The tendency for irreversible processes to generate entropy is the ultimate driving force for structure evolution in nature. In engineering, entropy production is often used as an indicator for loss of usable energy. In this study, we show that the analysis of entropy production patterns can provide insight into the diverse observations from experiments that investigate porous medium dissolution in imposed flow field. We first present a numerical scheme for the analysis of entropy production in dissolving porous media. Our scheme uses a greyscale digital model for chalk (an extremely fine grained rock), that was obtained using X-ray nanotomography. Greyscale models preserve structural heterogeneities with very high fidelity. We focussed on the coupling between two types of entropy production: the percolative entropy, generated by dissipating the kinetic energy of fluid flow, and the reactive entropy, originating from the consumption of chemical free energy. Their temporal patterns pinpoint three stages of microstructural evolution. We then showed that local mixing deteriorates fluid channelisation by reducing local variations of reactant concentration. We also showed that microstructural evolution can be sensitive to the initial transport heterogeneities, when the macroscopic flowrate is low. This dependence on flowrate indicates the need to resolve the structural features of a porous system when fluid residence time is long.

## Introduction

The production of entropy in irreversible processes drives the emergence and transformation of many structures in nature [1, 2]. Spatial and temporal patterns of entropy production can help us understand the diversity and the self-organisation inherent to many complex systems [3, 4]. In engineering, all loss of usable work can be monitored in units of entropy generation as a currency [5]. Reactive infiltration instability stems from positive feedback between the coupling of chemical reaction and mass transfer and induces the development of a wide variety of biotic and abiotic flow systems [6–10]. Predicting the development of flow systems in porous media is essential for many energy and environmental applications, such as geologic carbon storage [11], oil reservoir simulation [12], bioremediation [13] and *in situ* contaminant remediation [14]. Characterising the inherent heterogeneities of a porous material is important because infiltration instability can amplify transport heterogeneities indefinitely [15, 16]. It is thus desirable to use nondestructive, three dimensional imaging techniques, such as high resolution X-ray tomography [17–20], to obtain greyscale microstructure models of porous media [21, 22]. Because of the information preserved in these models, analysis of entropy production can be carried out with very high fidelity [17].

In this study, we treated a porous medium as a thermodynamic device that constantly dissipates the energy it receives from the environment. The evolution of the device’s internal structure is affected by its initial morphology and the energy input. The initial morphology dictates the pre-existing heterogeneities in the transport properties of the medium, while the energy input describes the different forms of energy that are dissipated by the device. Here we focus on the kinetic energy of fluid flow and the chemical energy of reactive solutes.

**Fig 1:**
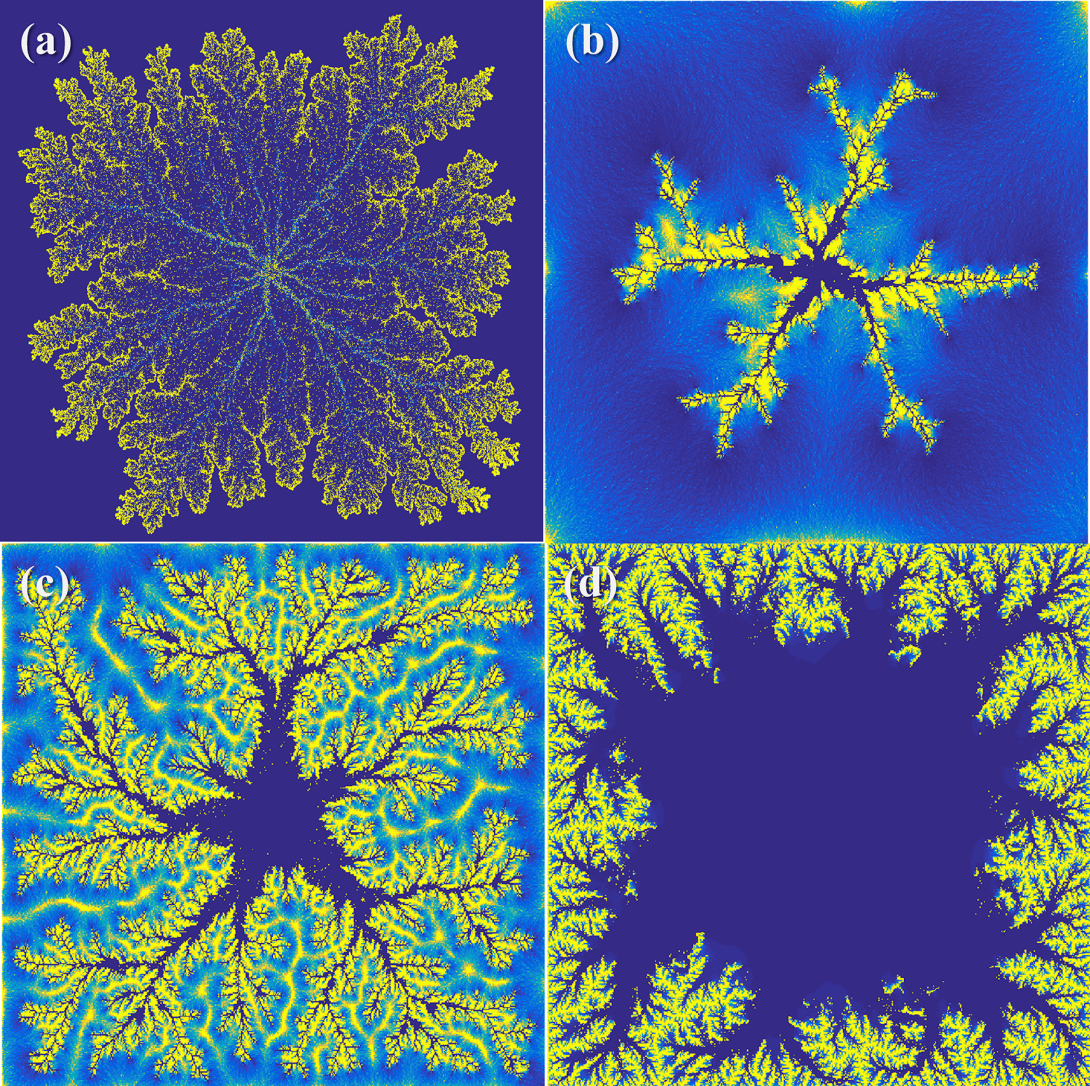
Example morphologies of simulated, developing flow systems dominated by infiltration instability. A dissolutive fluid is injected from the centre of the 2D domain, which consists of 4 million, i.e. 2000 by 2000, reacting units. The colours correspond to the apparent solid dissolution rate (yellow means greater rates). (a) a system where development is limited by fluid reactivity, i.e. fluid reaches equilibrium before it leaves each simulation domain; (b): a system where development is limited by solid availability, i.e. the solid depletes locally before the fluid reaches chemical equilibrium; (c) a system with a low injection rate compared with (d) a high injection rate.

Fig 1 shows four examples of a simulated, developing flow system, where a reactive fluid is injected at the centre of a dissolving 2D porous medium. With different properties of fluid flow, very different morphologies and patterns of entropy production can develop from precisely the same initial geometry. Experimentally, such a study can be almost impossible to perform because the initial geometry is inevitably destroyed after each experiment. Currently available manufacturing technology has not yet made it possible to reliably replicate natural geologic materials with identical geometry and local chemical homogeneity at the submicrometre level. In this study, we analysed numerically the effects of global fluid input on energy dissipation in a natural porous material with a fixed initial geometry. The geometric profile was obtained from a nanometre resolution tomogram of a chalk sample [23].

Chalk is a frequent host rock for drinking water aquifers, oil and gas reservoirs and because of its calcium carbonate composition, it serves as a useful model for a fast reacting rock. Chalk is dominated by the shields of coccolithophorids. These shields, called coccoliths, consist of nanometre to submicrometre sized single calcite crystals, that are irregular in size, shape and orientation [24]. Characterising the microstructure in chalk at the pore scale is difficult because of its extremely small grain size and its geometric complexity so chalk also serves as an excellent model for testing the resolution limits in our work. The complexities are encoded in the greyscale intensities in tomography reconstructions. They were preserved in the 3D images by avoiding simplification, which would usually arise from segmenting the reconstruction. We transferred the greyscale intensities to a material density representation, which maintains comparability between samples. Our aim was to present a mathematical scheme that uses the derived voxel level porosity in simulations of entropy production and to show: (i) that three stages of microstructural evolution during dissolution result in distinct temporal patterns in entropy generation, (ii) that local mixing deteriorates reaction front instability and (iii) that transport heterogeneities become more important when the macroscopic flowrate is low.

Reactive infiltration instability has been formulated mathematically by pioneers of geochemical transport modelling [6–9, 15, 16, 25–29]. This work aims to incorporate a high resolution greyscale nanotomography reconstruction of a natural porous material into an irreversibility analysis. This inclusion of realistic geometries is useful because of the sensitivity of the reaction front propagation to spatial heterogeneities [28]. Therefore, by fixing the initial geometry, our results allow us to unambiguously define the influence of global energy constraints on morphology evolution. It is also possible to put the much diversified and sometimes inconsistent experimental observations from real reactive percolation systems into a coherent big picture [30].

## Material and methods

We used a previously published static tomogram of Hod chalk (HC16) from the North Sea Basin, collected with the X-ray holotomography setup at the ID22 beamline at the European Synchrotron Research Facility (ESRF), in Grenoble, France [31]. This data set offers high spatial resolution and has been shown to be most amenable to the image processing algorithms developed by our team in a few related studies [32–37]. The data were reconstructed from 1999 radiographs (360° rotation, 0.5 s exposure) at 100 nm voxel resolution using the holotomography reconstruction method [23, 31] and processed as described in Bruns et al. [35, 36]. The goal of the processing routine was to generate a greyscale volume image where variations in voxel intensity could be related to local material density, i.e. greyscale variations resulted from partial volume effects and not from signal blur, noise or artefacts. The average intensities of these phases are initially unknown so we used a seven phase Gaussian mixture model to identify the most likely intensities for the chalk and void phases, resulting in a macroscopic porosity of 0.22 for the 1350×1350×1514 voxel reconstruction. From this reconstruction, a subvolume sample of 60×60×300 voxels (6×6×30 μm^3^) were chosen and subjected to a general dissolution reaction, which we can write for Material A as: A (solid) ⇔ A (aqueous).

We adopted the model framework of Yang *et al*. [32–34, 37]. The mathematical scheme is designed to treat the assemblage of greyscale voxels as a network of ideal chemical reactors. Any structural feature that is smaller than a voxel size is homogenized in a greyscale tomographic data set. Nevertheless, greyscale data contain more microstructure information than binarised (segmented) data. This is because the voxel intensity that translates to porosity through the Gaussian mixture model is a function of X-ray absorbance. A porosity value between 0 and 1 indicates that a real structure exists inside that voxel and the magnitude of the intensity reflects the density of the material. In a 32-bit greyscale data set, 2^32^ different sub-voxel possibilities can be registered. In comparison, voxels in a segmented tomogram can only take two values.

The advantage of preserving information of structure smaller than a voxel in numerical simulation is meaningful for two reasons. First, chalk is a rock with very fine grains, whose features are difficult to be fully resolved. Yet small heterogeneities can be important in distorting the morphology of migrating reaction front and as a result, shaping the porous structure development. Having an alternative mathematical scheme, which offers the possibility to import these features into numerical simulation, can be very useful. Second, governing equations that are derived from first principles, e.g. the NS equations, can only be applied to void space with explicit surface boundaries. These equations are better grounded on physics than the phenomenological equations that describe chemical reactor behaviours. However, in pursue of the overall physical realism, one needs to consider both the governing equations and the geometry to start with. It remains to be seen whether the gain in the physical soundness can compensate for the significant loss of microstructural information when a segmented initial geometry is used.

The following equations quantify voxel behaviour–each voxel is considered phenomenologically as a set of ideal chemical reactors. These reactors are the elementary units in the numerical simulation that follows. I.e. the scheme is inherently discrete. The equations used in the simulation are summarised below. In the model, all physical quantities were first normalised to reduce the total number of parameters:

$$q=\frac{Q}{Q_{0}},\;l_{n}=\frac{l}{L_{ref}},\;p=\frac{P}{P_{ref}}\;\mathrm{and}\;C=\frac{C_{A,eq}-C_{A}}{C_{A,eq}-C_{A,inj}},$$ (1)

where *Q* represents the voxel level volumetric flowrate (m^3^/s), *l*, the voxel size (nm) and *P*, the pressure (Pa). *Q*_*0*_, *L*_*ref*_ and *P*_*ref*_ represent the reference values, which can be chosen arbitrarily. In this study, they were given the following values:

$$Q_{0}={\left[-\frac{\left(\mu_{A}^{0}+RT\right)\left(C_{A,eq}-C_{A,inj}\right)}{k_{A0}{}^{1/3}\cdot \mu \left(\varphi_{\exp}/k_{\exp}\right)}\right]}^{1.5},$$ (2)

$$L_{ref}=\sqrt{-\frac{\left(\mu_{A}^{0}+RT\right)\left(C_{A,eq}-C_{A,inj}\right)}{k_{A0}\cdot \mu \left(\varphi_{\exp}/k_{\exp}\right)}},\;\mathrm{and}$$ (3)

$$P_{ref}=-\left(\mu_{A}^{0}+RT\right)\left(C_{A,eq}-C_{A,inj}\right),$$ (4)

where $\mu_{A}^{0}$ represents the reference chemical potential of A (kJ/mol), *R*, the universal gas constant (kJ/mol·K), *T*, the temperature (K), *μ*, viscosity of the fluid (Pa·s), *φ*_exp_ and *k*_exp_, the experimentally measured porosity and permeability (m^2^) of the sample [23], and *k*_*A0*_, the apparent first order rate constant of the dissolution reaction (s^-1^), defined as:

$$k_{A0}=\frac{r_{A0}/ssa}{C_{0}\left(C_{A,eq}-C_{A,inj}\right)},$$ (5)

where *ssa* represents the voxel level specific surface area (m^2^/m^3^) and *C*_*A*_, the concentration of A at the exit of a voxel (mol/m^3^). *C*_*A*,*eq*_ and *C*_*A*,*inj*_ represent the equilibrium concentration and the concentration of A in the injection fluid (mol/m^3^). *C*_*0*_ represents the dimensionless concentration at the inlet of a voxel (i.e., corresponding to *C*_*A0*_) and *r*_*A0*_ represents the dissolution rate (mol/m^2^·s) where *C = C*_*0*_.

The pressure drop across a voxel is simplified to follow Darcy’s law:

$$q=-\left[\left(\frac{P_{ref}L_{ref}}{\mu \cdot Q_{0}}\right)\left(\frac{k_{\exp}}{\varphi_{\exp}}\right)\right]\cdot \left(l_{n}\varphi^{2}\right)\cdot \Delta p,$$ (6)

in which *φ* represents the voxel level porosity. The voxel level tortuosity and permeability are expanded around zero with respect to the empty volume in each voxel. The truncation error is expected to decrease with the voxel size, *l*_*n*_.

For each voxel, the continuity equation for an incompressible fluid applies at the center:

$$\sum_{i=1}^{6}q_{i}+q_{s}=0.$$ (7)

where *q*_*i*_ is the volumetric flow from the six neighbouring voxels and *q*_s_, from the environment.

The macroscopic fluid distribution was determined, based on the permeability tensor:

$$Κ=NxKxNx^{T}+NyKyNy^{T}+NzKzNz^{T},$$ (8)

where **Nx, Ny** and **Nz** are nodal matrices along the three Cartesian axes and **Kx, Ky** and **Kz** are diagonal matrices containing voxel level permeability values. The pressure field can be calculated using

$$Q_{S}=-Κp,$$ (9)

where **Q**_**s**_ and **p** represent vectors that describe the global constraints on the system and its pressure field.

In each voxel, the chemical conversion in the subvolume with complete mixing was calculated to be:

$$-\sum_{i}q_{i}C_{0i}+\left(1+Da\right)\cdot qC=0,$$ (10)

in which *i* indicates the summation over all inlets of reactive fluid and the Damköhler number, *Da*, is defined as:

$$Da=\left(\frac{r_{A0}}{C_{0}\left(C_{A,eq}-C_{A,inj}\right)}\right)\cdot \left(\frac{L_{ref}^{3}}{Q_{0}}\right)\cdot \eta \cdot l_{n}^{3}\cdot \left(\frac{ssa}{q}\right),$$ (11)

where *η* represents the portion of a voxel within which reactants and products are completely mixed. The conversion in the nonmixing subvolume was calculated from

$$-C_{0}+e^{Da}\cdot C=0,$$ (12)

where the reactor volume in *Da* takes a different value:

$$Da=\left(\frac{r_{A0}}{C_{0}\left(C_{A,eq}-C_{A,inj}\right)}\right)\cdot \left(\frac{L_{ref}^{3}}{Q_{0}}\right)\cdot \left(\frac{1-\eta }{3}\right)\cdot l_{n}^{3}\cdot \left(\frac{ssa}{q}\right).$$ (13)

The dissolution reaction is assumed to be much slower than establishing the velocity profile and the voxel level porosities was updated according to:

(η⋅dφ)i=q(C0−C)⋅(CA,eq−CA,injρ/M)⋅(Q0Lref3)⋅(dtln3). (14)

It is worth emphasising that the aforementioned modules–the governing equations for fluid flow and for solute transport (e.g., Eqs 6–9), the fidelity of the initial geometry (the tomographic dataset) as well as the kinetics of mineral dissolution (the linear rate law used in this study to derive Eqs 10–13)–can be replaced by their counterparts. And more accurate physical representations of these modules will produce more realistic simulation results. We chose to use these equations because they allowed us to put into the context of a physically realistic rock microstructure the following general system behaviours. First, given a constant macroscopic flowrate, fluid tends to be distributed according to local permeability differences, and more porous materials are more permeable. Second, the rate of mineral dissolution relies on the chemical affinity of the dissolution reaction, which in turn relies on the residence time of the stream. Third, the presence of inherent transport heterogeneities distorts the morphology of migrating dissolution front. To give a qualitative description of the consequences stemming from these behaviours, we define the following entropy production terms. Percolative entropy generation (S_gen, per_) is the rate of entropy production in flow with friction [5]. It is proportional to the volumetric flowrate and the pressure drop in each voxel and is calculated using:

$$\left(\frac{T\cdot {\dot{S}}_{gen,per}}{Q_{0}\cdot P_{ref}}\right)=\frac{q^{2}}{l_{n}\varphi^{2}}/\left[\left(\frac{P_{ref}L_{ref}}{\mu \cdot Q_{0}}\right)\left(\frac{k_{\exp}}{\varphi_{\exp}}\right)\right].$$ (15)

The reactive and mixing entropies were not distinguished because the chemical conversion in each reactor is inherently determined by its extent of mixing. The reactive entropy term is therefore calculated as the change in Gibbs free energy on the voxel scale:

S˙gen,rxn=−(μA0T+R)(CA,eq−CA,inj)Q0⋅q⋅(C0−C). (16)

## Results

### Three stages of microstructural evolution that show distinct patterns of entropy generation

**Fig 2:**
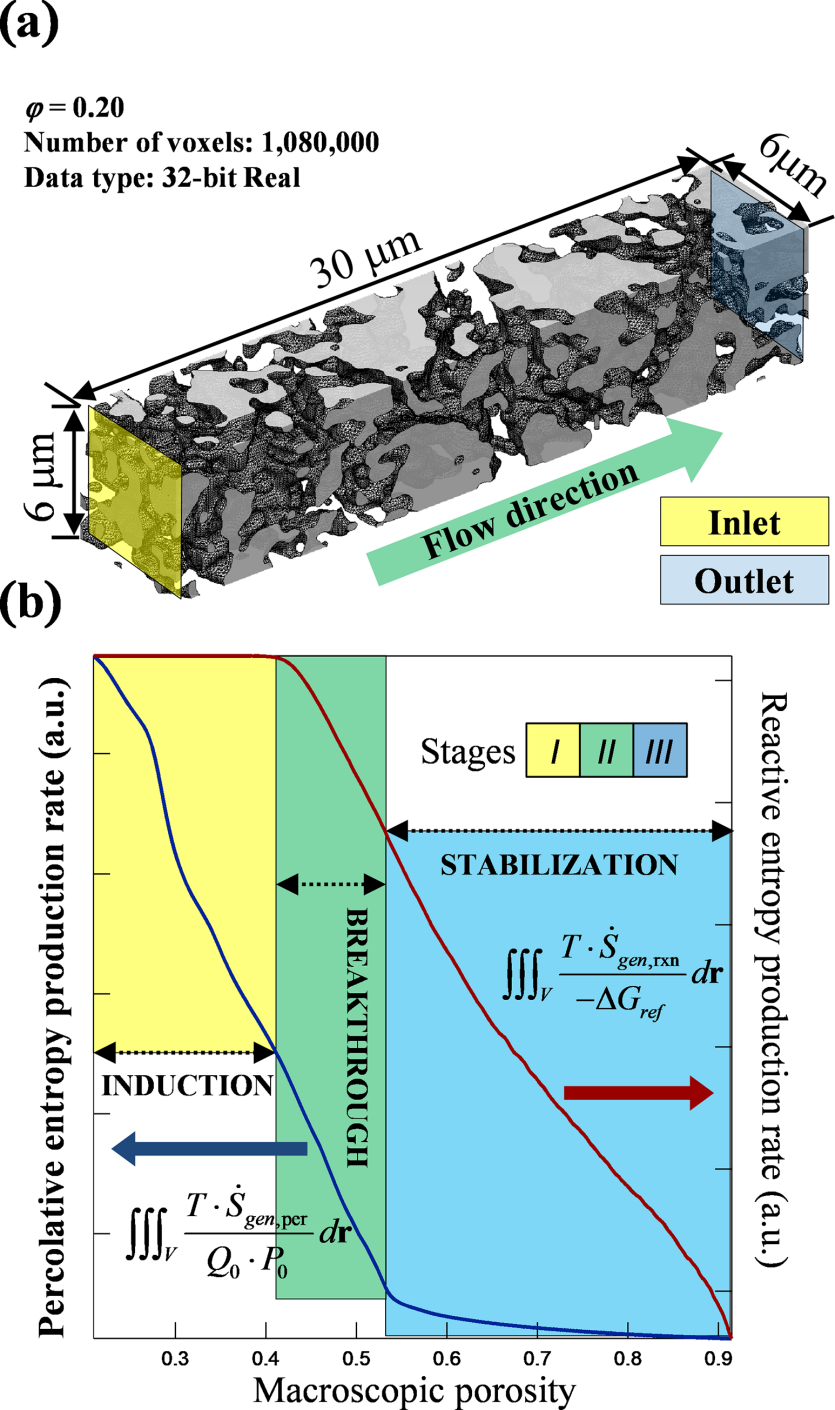
Temporal pattern of percolative and reactive entropy production based on a 32 bit greyscale tomography reconstruction of chalk. (a) Perspective view of the simulation domain where the initial porosity is 0.20; (b) temporal patterns of entropy generation divide the microstructural evolution into three stages: induction, breakthrough and stabilisation. The volume averaged, i.e. macroscopic, porosity is used as a measure for the overall reaction progress. The blue curve shows the evolution of *S*_*P*_, the percolative entropy production, and corresponds to the left axis; the red curve shows *S*_*R*_ and corresponds to the right axis (indicated by arrows). The dimensionless quantities are normalised with respect to combinations of reference temperature (T), flowrate (*Q*_0_), pressure (P_0_) and Gibbs free energy (*-ΔG*_*ref*_). The equations are marked next to the plots. The two curves are rescaled using their initial and final values.

Fig 2 shows the initial geometry for the porous medium used in this study and the temporal patterns of entropy generation by the various mechanisms. The geometry is modelled using a greyscale tomography reconstruction that realistically represents the microstructure of a 6 x 6 x 30 μm^3^ sample of North Sea Basin chalk, with a macroscopic, i.e. volume averaged, porosity of 0.20. The volume consists of 1.08×10^6^ voxels (100^3^ nm^3^ each) and the porosity of each voxel is represented by a 32 bit real number. We simulated an injection of reactive fluid from the inlet (yellow plane in Fig 2A) to the outlet (blue plane). The normalized percolative entropy, *S*_*P*_, is an integral of entropy generated over the simulation domain for overcoming fluid friction and serves as a measure of kinetic energy dissipation. The reactive entropy, *S*_*R*_, is calculated based on changes of Gibbs free energy in each and every voxel and is a measure of chemical energy dissipation.

**Fig 3:**
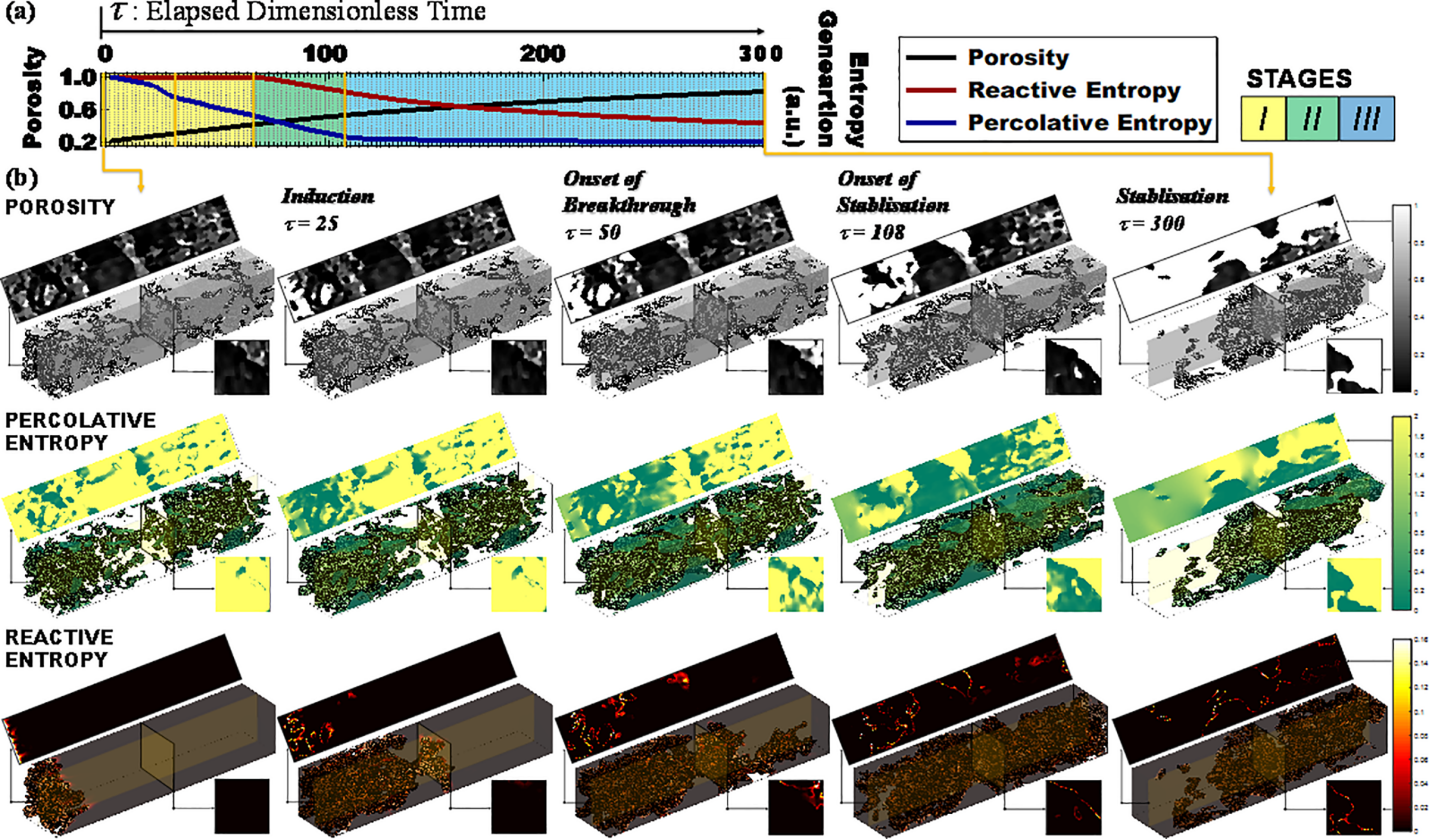
Patterns of entropy production during microstructural evolution. (a) Temporal pattern. The black, blue and red curves show the evolution of macroscopic porosity, percolative entropy and reactive entropy production rate. The three stages of structure development are colour-shaded: yellow–induction; green–breakthrough; blue–stabilisation. Five representative instants are chosen from the timeline (marked by yellow vertical lines in a) and visualised in (b). (b) Spatial patterns–porosity distribution (grey, first row), percolative entropy (yellow-blue, second row) and reactive entropy (red-black, third row). The 3D perspective views are isosurface drawn at a handpicked value of the corresponding quantity. The rectangular and square images (sheets above and below each three-dimensional figure) are cross sections of the corresponding quantities at the middle of the radial and the axial directions (indicated by semi-transparent boxes imposed on the perspective views). The colour bars indicate the dimensionless, numerical values of the corresponding quantity. The pattern of percolative entropy generation inverses after breakthrough because of fluid channelling. The spatial patterns of reactive entropy visualise the dissolving regions in the pore structure.

The temporal patterns of percolative entropy, *S*_*P*_, and reactive entropy, *S*_*R*_, showed three stages during the microstructure evolution: induction, breakthrough and stabilisation (Fig 2B). During the induction phase, *S*_*R*_ remained on a plateau because the residence time was sufficiently long that the fluid reactivity was depleted, i.e. it reached chemical equilibrium before arriving at the outlet. By monitoring the effluent composition, one might naïvely conclude that the system had reached steady state. However, tracking *S*_*P*_ revealed the system dynamics. Percolative entropy generation decreased nonlinearly because mineral dissolution led to the development of a dominant flow path within the complex geometry. This development started from the inlet and the dissolution front in that pathway advanced gradually downstream, preferentially removing solid material in the more porous voxels (Fig 3, porosity). This biased removal is characteristic of reactive infiltration instability, which amplifies local differences in permeability.

The arrival of the reaction front at the outlet (Fig 3, reactive entropy at dimensionless time, *τ* = 50) marked the start of the breakthrough stage. During breakthrough, a significant increase in macroscopic permeability was accompanied by a shift in the spatial pattern of percolative entropy generation. The newly developed major flow path connected the fluid inlet and outlet, thereby channelling the fluid. This minimizes the energy dissipation for overcoming flow resistance. Before this change took place, regions with lower porosity generated more percolative entropy (Fig 3, porosity and percolative entropy, *τ* = 50). After breakthrough, this pattern reversed as a result of fluid channelling (Fig 3, *τ* = 108 and onward). It is noted that the macroscopic flowrate was kept constant throughout the microstructure development. When a different boundary condition applies, e.g. if a constant pressure difference is imposed, the overall flux should increase with the rising permeability, and more viscous entropy would have been produced. And this difference would have been reflected in the slope of the *S*_*P*_ curve near the inflection point. Meanwhile, *S*_*R*_ began to decrease as a result of shortened residence time. The last inflection point in *S*_*P*_ marked the end of breakthrough and the beginning of a stabilisation phase. During stabilisation, *S*_*P*_ was dominated by the expansion of the major flow path and decreased gradually until all solid was depleted. Reactive entropy generation was limited to regions where sharp gradients of porosity were observed (Fig 3, *τ* = 300). These regions can be physically interpreted as solid-fluid interfaces and this change in the spatial pattern marked a transition from advection driven reaction pattern to one that is dominated by interfacial interactions.

### Local mixing deteriorates fluid channelisation

Mixing can be considered the net effect of solute transport. Different transport mechanisms produce different mixing patterns. For example, convection preserves the composition of the bulk flow while diffusion is driven by concentration gradients and tends to smear out spatial variations in the solute distribution. Hence, diffusion enhances mixing whereas convection does not. In this study, we did not distinguish among transport mechanisms. Instead, we analysed the net effect of mixing on the chemical conversion of reactants. This analysis was done by adjusting the contact pattern between reactant and product at the voxel level. A dimensionless parameter, η, was used for this purpose. η represents a continuous function that varies between 0 and 1. When η = 0, there is no mixing between reactant and product as a reaction takes place; when η = 1, reaction happens only when the reactant and the product are completely mixed. A simplistic analogy can be drawn between η and the Péclet number (*Pe*) on the voxel level. η close to 1 corresponds to a small *Pe*, *i*.*e*., a transport mechanism that enhances mixing (e.g. molecular diffusion) dominates. Similarly, η close to 0 corresponds to a large *Pe*. The caveat of adopting η is that one cannot predict precisely the system behaviour. Instead, η allows bracketing the possible outcomes of a real system by two extreme cases (η = 0 and 1). Assigning a single value of η to all voxels in a dataset also ignores local heterogeneity in the strength of molecular transport, which might not be appropriate when the scale of the system is large. Nevertheless, we consider η to be a general descriptor of mixing phenomena in this study because it is not limited to any specific combination of transport mechanisms and because of the moderate scale of the simulation domain.

**Fig 4:**
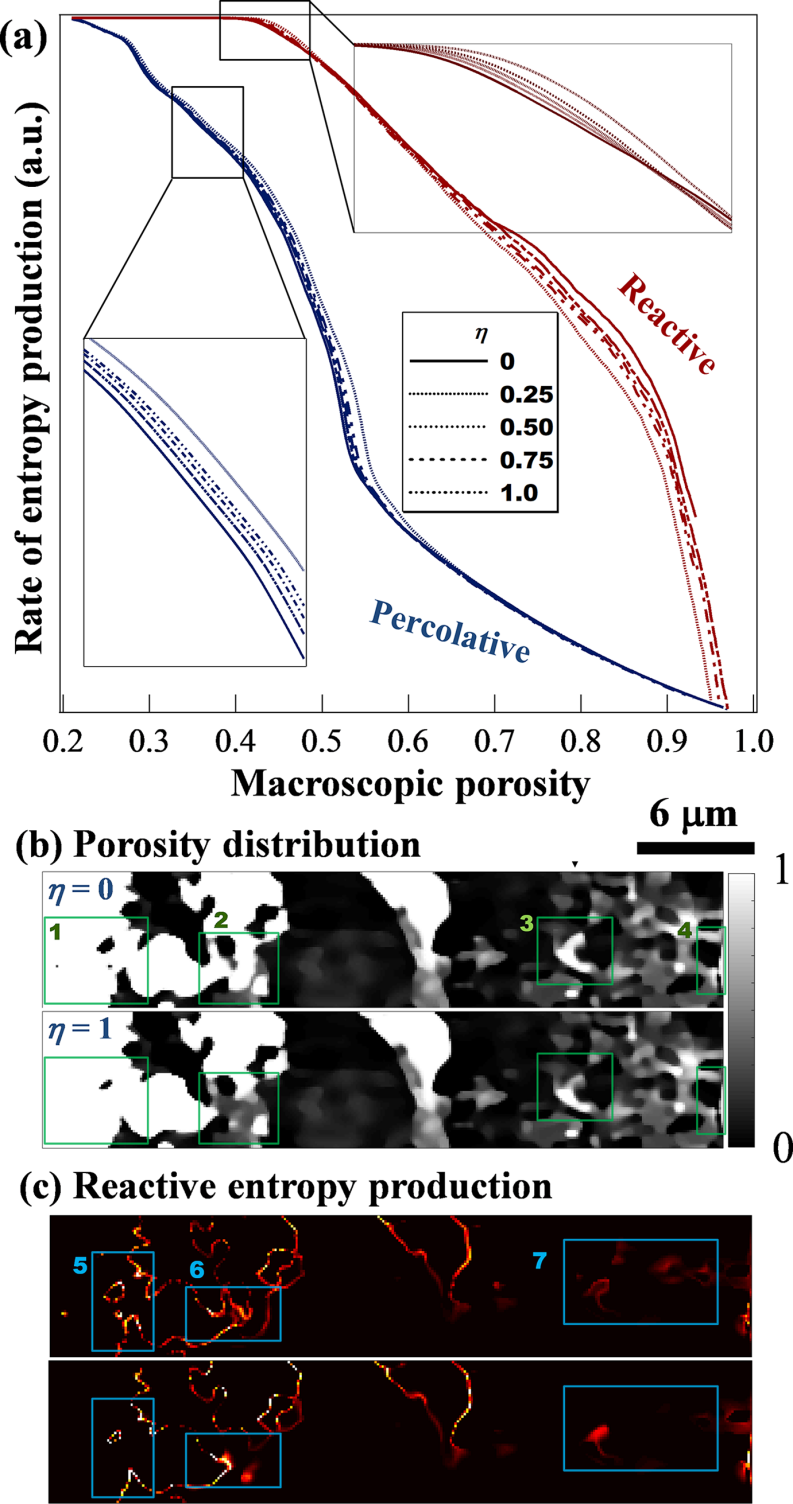
Impact of voxel level mixing on entropy production. η = 1 corresponds to complete mixing and η = 0, to no mixing between reactant and product. (a) Mixing effect on the temporal patterns of entropy generation. Percolative entropy was affected monotonically by mixing while reactive entropy was affected by two counteracting effects. (b) Cross sections of porosity distribution. The solid-liquid interface is sharpened near the fluid inlet, showing a rapid spatial transition from pure solid to pure fluid within a few voxels. (c) Distribution of reactive entropy production shows highly localised dissolving regions. Brighter means more dissolution. Mixing leads to a longer residence time and higher surface smoothness. Macroscopic porosity = 0.5 in (b) and (c).

Mixing protracts the development of the major flow path and delays the occurrence of breakthrough (Fig 4A). This delay is because mixing counteracts the instability that drives the morphing of the system. The evolution of the microstructure, especially the development of the major flow path, is induced by infiltration instability. This instability provides a positive feedback between mass transfer and mineral dissolution. The net observable effect is autocatalytic, i.e. flow and reaction enhance each other locally. Mixing introduces a negative feedback to this chain of coupling by reducing the reactant concentration and therefore the rate of the dissolution. This negative feedback can only be observed when the reaction rate increases monotonically with chemical affinity. This prerequisite is met by a wide range of geochemical reactions that follow a transition state theory (TST) rate law.

The effect of mixing on *S*_*P*_ escalates as the main flow path develops toward the outlet, reaching a maximum during the breakthrough stage. It then diminishes rapidly during stabilisation. This variation can be attributed to the sensitivity of the percolation entropy generation to the geometric complexity of a porous medium before the development of a main flow path. This flow path advanced faster without mixing, hence *S*_*P*_ was low when η was small. For example, for a volume with average porosity of 0.53, a 43% difference between η = 0 and 1 was observed for the mechanical energy dissipation, suggesting that more energy was needed to drive the same amount of fluid through an evolving microstructure. After breakthrough, the pressure drop was determined by the permeability of the major flow path and *S*_*P*_ became insensitive to mixing in the solid residual.

Fig 4B and 4C show cross sections (*x* = 30 μm) of the microstructure and the corresponding spatial patterns of reactive entropy generation in two limiting cases of micromixing. Because the impact of *η* is monotonic on *S*_*P*_ (Fig 4A), one could expect that the porosity distribution resembles an interpolation of the two images at an intermediate micromixing (*η* = 0.5). Regions of interest (ROIs) are highlighted with rectangular boxes. The temporal patterns of *S*_*R*_ suggest that, with decreased mixing, the reactive fluid becomes more penetrating and the reaction front reaches the outlet with less solid dissolved (upper right inset in Fig 4A). This effect is manifested in Fig 4B, given the same overall porosity, stronger mixing (η = 1) results in more thorough dissolution of the upstream materials (ROI1) but with a less developed pore structures downstream (ROIs 2–4). As a consequence, mixing led to increased residence time, leaving fewer voxels partially filled with solid.

These two aspects exerted opposite influences on reactive entropy generation. Long residence time enhances the chemical conversion and thus increases *S*_*R*_. Meanwhile, a decrease in the number of intermediate grey voxels can be physically interpreted as generation of a smoother surface on the material, which leads to a drop in reactive surface area and hence, *S*_*R*_. These counteracting factors result in a complex temporal pattern of entropy generation. Before breakthrough, residence time dominated the chemical conversion. A system with better mixing dissipates more chemical energy. After breakthrough, the residence time quickly converged to a single value that was determined by the major flow path. Therefore, a system with less mixing produces more reactive entropy than a well-mixed system. Fig 4A shows this transition between these cases: the apparent insensitivity of *S*_*R*_ to mixing during the breakthrough stage is actually a superposition of two contradicting effects. During the stabilisation stage, *S*_*R*_ was greater for systems with a “rougher” surface (in a porosity distribution image, a sharp interface is manifested by a rapid transition from black to white pixels, in the absence of transitional, grey features; in a reactive entropy production map, a sharp interface is indicated by bright, thin lines).

Fig 4C shows that while the overall *S*_*R*_ was similar for both systems at *φ* = 0.5, less mixing led to weaker but more dispersed entropy hotspots (ROIs 5–7). In contrast, when *η* = 1 the system showed sharp and bright entropy hotspots along the interfaces upstream but far less so downstream. In general, more mechanical energy is needed to drive the evolution of a porous medium toward complete dissolution when mixing is strong. We would like to emphasize that the overall entropy production is not conservative–because the two types of entropy are not produced from the same type of energy input. However, their patterns do correlate and can be interpreted from the mass transfer perspective. Both the porosity distribution and the spatial distribution of reactive entropy production are ultimately affected by the morphology of solid-fluid interface, where the rock erodes. But this effect is not instantaneous and will only become clear as time elapses. The impact of local mixing is to “blur” the solid-liquid interface. This blurring stems from the tendency of mixing that reduces the gradient of solute concentration (and this effect is instantaneous and observable within the same time step).

### Greater flow rate reduces the impact of initial heterogeneities

**Fig 5:**
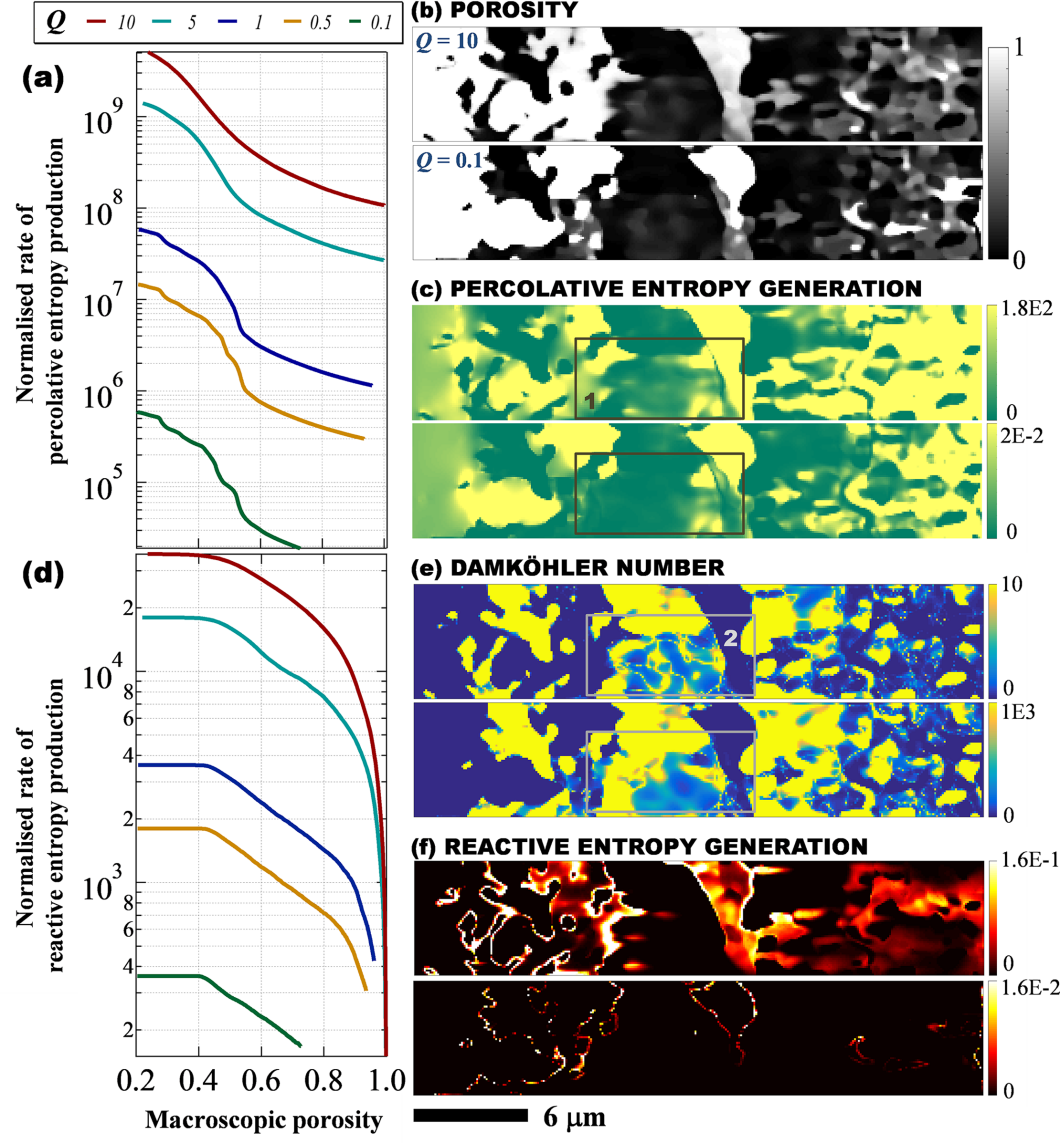
Effects of macroscopic flow rate, *Q*, on microstructural evolution. (a) Temporal patterns of percolative entropy generation. (b) Cross sections of porosity distribution at different *Q*. (c) Spatial patterns of percolative entropy generation with regions of interest (ROI) showing entropy hotspots in a low porosity subvolume at high fluid throughput. (d) Temporal patterns of reactive entropy generation. (e) Local Damköhler number with ROI 2 showing distinct spatial patterns of *Da* in a low porosity subvolume. (f) Spatial patterns of reactive entropy generation. In (a) and (d), the dimensionless quantities (*S*_*P*_ and *S*_*R*_) are normalised with respect to the same combination of reference quantities, i.e. reference temperature, flowrate, pressure and Gibbs free energy (see also Fig 2B). In (b), (c), (e) and (f), all cross sections were made when macroscopic porosity = 0.5, with the upper image showing *Q* = 10 and the lower one, *Q* = 0.1.

In this study, porous media are considered thermodynamic devices that dissipate the energy they receive from the environment. It is thus of interest to investigate the effects of a global constraint, i.e. the total amount of energy that a medium receives, on the microstructural evolution. We used the macroscopic flow rate as a measure of this global constraint because it reflects both the energy requirement for overcoming the resistance to the fluid flow and the amount of chemical reactant input given the same solvent composition. Given the size of a simulation domain, a greater flow rate indicates a shorter residence time for the medium to digest fluid reactivity. Fig 5 shows the impact of the macroscopic dimensionless flowrate, *Q*, on the temporal and spatial patterns of entropy generation. Both percolative and reactive entropies scale with the flow rate because with greater throughput, the medium received more mechanical and chemical energy. *S*_*P*_ increases with *Q* because more energy is required to drive a larger volume of flow through the same microstructure. The temporal pattern of *S*_*P*_ is most sensitive to the geometric complexity when *Q* is low, while with greater *Q*, the system resembles a homogeneous medium, for which the various stages of structural evolution are not distinct. For example, in Fig 5A, the last inflection point of *S*_*P*_ can be easily identified for *Q* = 0.1 (at φ = 0.53) but not for *Q* = 10 (at a φ less than 0.4). Close examination of the geometric cross sections (Fig 5B) shows that high fluid throughput results in greater surface roughness (manifested by a higher number of partially filled voxels). In contrast, the cross section of *Q* = 0.1 shows that the original heterogeneities have been amplified, leaving sharp interfaces and a more thoroughly dissolved upstream geometry. Fig 5C shows that the channelling effect is insignificant for *Q* = 10 and many less porous locations are percolative entropy hotspots (ROI 1).

The impact of the global flow rate is also evident in the patterns of reactive entropy production. Fig 5D shows that the temporal pattern of *S*_*R*_ can also be divided into three stages. In the first stage, *S*_*R*_ is limited by the fluid reactivity. Given reactant concentration at the inlet, the available chemical energy scales with flow rate. The second stage shows a gradual decrease of *S*_*R*_ as the major flow path expands and the fluid residence time shortens. The last stage features a quick drop of *S*_*R*_, which stems from the depletion of solid reactivity as the macroscopic porosity approaches unity, i.e. complete dissolution. When less reactant is available, solid reactivity depletion is delayed and vice versa. When *Q* = 10, the third stage of *S*_*R*_ begins before the macroscopic porosity reaches 0.8, while for *Q* = 2.0 this change takes place at approximately φ = 0.9. A visualisation of local Damköhler number (Fig 5E, Eqs 11 & 13) shows that greater fluid throughput drives the reactants into less permeable regions and thus enlarges the sample subvolume that is available for chemical reactions (ROI 2). However, chemical conversion in these voxels is low because of the short residence time, leaving many partially dissolved voxels behind. The spatial pattern of *S*_*R*_ (Fig 5F) clearly shows that the reaction is more convection driven and dispersive when *Q* = 10 and is more interface dominant when *Q* = 0.1.

The effects of the global constraint on microstructural evolution have two important implications. First, the amplification of transport heterogeneity during structural morphing is *Q* dependent. The distinguishability of different stages in the temporal entropy generation is a measure of system sensitivity to the initial geometry. Fig 5 shows that this distinguishability depends heavily on the global constraint, which implies that the reactive infiltration instability is important in big systems where the fluid input can be considered small and local. In contrast, the prediction of system evolution with a large *Q* requires lower resolving ability of the initial geometry. Second, percolative entropy generation is closely related to the macroscopic permeability of a porous medium. When the evolution of a system needs to be accounted for, a representative elementary volume might have to be defined accordingly, not only regarding the nature of the porous material but also the specific petrophysical parameter and the environment that controls the global constraint, e.g. flow rate.

## Discussion

Irreversibility analysis based on greyscale tomography images provides unprecedented insights into the structural development in a natural, microfluidic system. Assuming chemical homogeneity, the evolution of a system is controlled by only four dimensionless parameters: voxel level porosity (φ), dimensionless voxel size (*l*_*n*_), extent of regional mixing (η) and the global constraint (measured by *Q*). φ and *l*_*n*_ are determined by the tomographic data but η and *Q* can vary, depending on the nature of the porous material and the operational conditions of core flooding. This variability can help us decouple the effects of geometric complexity from those of other factors by conducting numerical experiments with a fixed initial geometry. This method allows us to revisit many experimental observations and put the results into a more coherent and big picture.

A defining moment during the instability induced microstructural evolution is the fluid breakthrough. This is the moment when the location where most percolative entropy is generated changes from the porous portion of the medium (partially occupied by solid) to the channels (fully occupied by fluid), *e*.*g*. Fig 3, *S*_*P*_ pattern at *τ* = 50 vs *τ* = 108. This transition leads to distinct system behaviours, which in the past have been interpreted differently and given various names. Before breakthrough, the major flow path develops from the reactive fluid inlet and slowly advances downstream. Because of the system’s tendency to amplify transport heterogeneity, this path development is usually accompanied by the preferential removal of the more permeable or more reactive materials. For example, microcrystalline and concomitant particles have been observed to dissolve preferentially during the early stage of acidic fluid percolation in limestone [38, 39]. The sharpening of solid-liquid interfaces after breakthrough, most clearly shown in the spatial patterns of reactive entropy generation (e.g. Fig 3, *τ* = 300 and Fig 5F), has also been observed by registering boundary geometry [30, 40]. This interface focussed reaction pattern that accompanies the expansion of the major flow path has been referred to as sparitic dissolution [38], interface smoothing [39], surface/transport control [41] and heterogeneous dissolution [42]. In contrast, the convective and dispersive pattern before breakthrough has been referred to as reaction control or uniform/homogeneous dissolution [30]. The homogeneous/heterogeneous categorisation is particularly informative because it describes the spatial patterns of structural evolution and also implies that as *Q* approaches infinity, the effect of inherent geometric heterogeneity on morphology development would vanish (Fig 5A), i.e. a porous medium would evolve as if perfectly homogeneous. It is worth emphasising that homogeneous and heterogeneous dissolution patterns can coexist and are separated spatially by the advancing front of the developing major flow path (e.g. Fig 3., *S*_*R*_ pattern at *τ* = 108 and Fig 5F, *Q* = 10). The breakthrough event, marked by the last inflection point in the temporal *S*_*P*_ pattern, has been documented experimentally as a transition regime [42]. Physically, breakthrough means connection between the percolation inlet and outlet by the newly developed major flow pathway (Fig 3). As a result, the macroscopic permeability increases sharply with a minor change in porosity [42]; the pore connectivity increases [38, 39] as small pores are interconnected through the major flow path; the volume averaged tortuosity decreases [42] because of the shortened residence time and both the effective porosity [43], i.e. the porosity in the convective subvolume of a sample, and the effective hydraulic radius increase [42] because the fluid is channelled into the major flow path.

**Fig 6:**
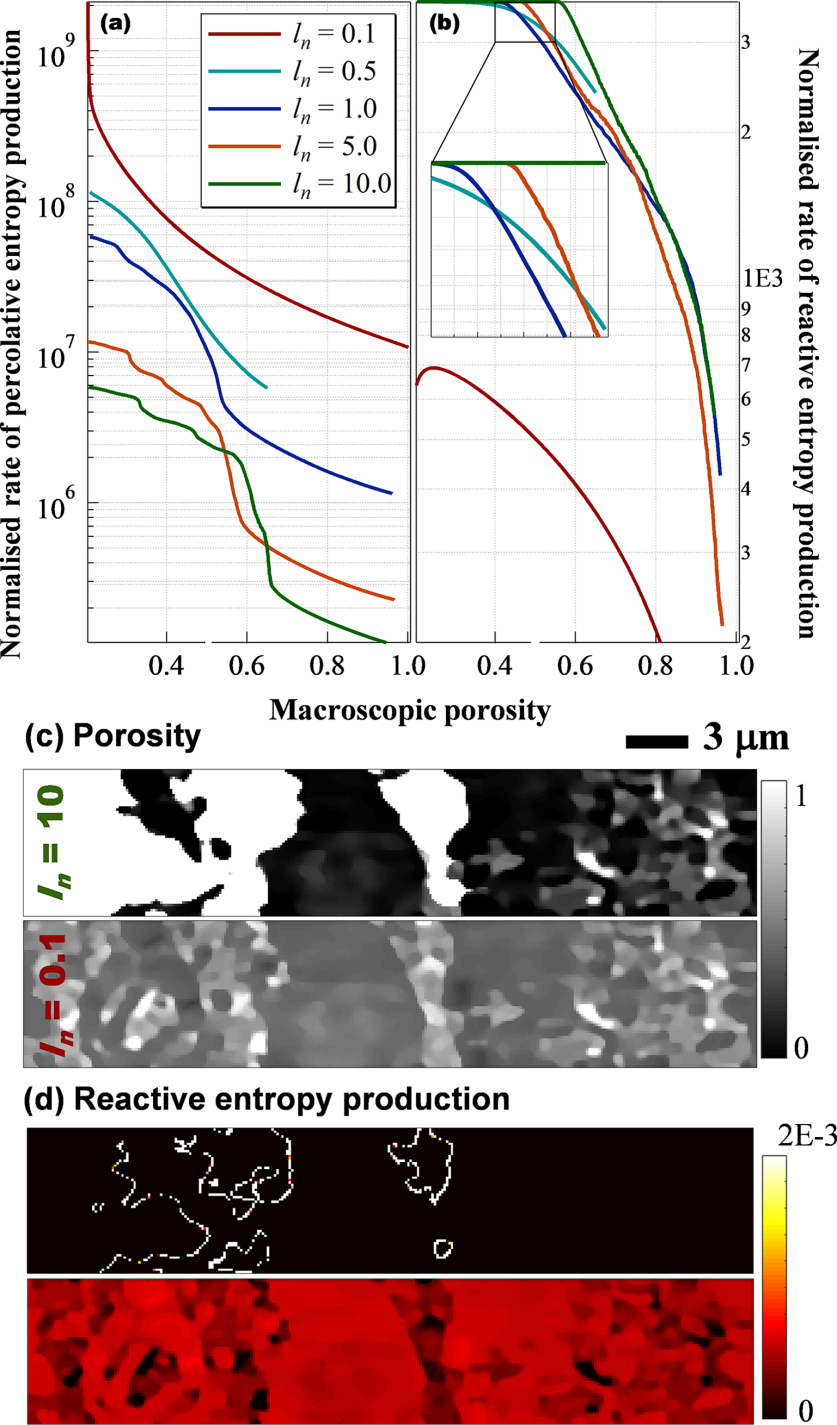
Effect of system size on entropy generation. System size is varied by changing the dimensionless voxel size (*l*_*n*_) while keeping the geometry constant. A smaller *l*_*n*_ corresponds to a system with less solid material and, given the same fluid throughput, greater fluid residence time. (a) and (b) temporal patterns of entropy production. (c) and (d) cross sections of porosity and entropy generation when macroscopic porosity reaches 0.5. A smaller system dissolves more homogeneously, consistent with experimental observations.

The temporal pattern of *S*_*R*_ helps reconcile inconsistencies between experimental observations based on solution chemistry analysis. The value of *S*_*R*_ reflects the overall dissipation of chemical free energy entering the system and can thus be related to the outlet concentrations of reactants (e.g., pH) or products (e.g., metal cations). Percolation experiments might cover different stages of the *S*_*R*_ evolution and observe inconsistent trends in outlet concentration and, as a derivative, reactive surface area. This apparent contradiction can be best interpreted by the relative reactivity between the fluid and the solid. Fig 6 shows the effects of *l*_*n*_, used as a measure of system size, on entropy generation patterns. Small *l*_*n*_ corresponds to a medium with the same geometric complexity but smaller in scale (and thus containing less solid material given the same porosity). If percolation is initially limited by fluid reactivity (Fig 6B, *l*_*n*_ = 0.5, 1.0, 5.0 and 10.0) the effluent would be saturated with dissolving minerals [44]. This quasi steady state corresponds to the initial plateau in reactive entropy generation. As the pore structure develops, the outlet concentration of dissolution products decreases exponentially as a result of shortened residence time [38, 41, 42]. The concentration can eventually drop to zero as the reaction becomes limited by solid availability (e.g. as φ approaches 1, when the microstructure dissolve completely). However, if the percolation is initially limited by solid reactivity, as is very often seen in aluminosilicate dissolution [45, 46], it is possible for the reactive surface area to increase over time, as more pores open, enabling interfacial contact (Fig 6B, initial *S*_*R*_ increase for *l*_*n*_ = 0.1). If percolation starts from the stabilisation stage with pre-existing major flow paths (e.g. fractures in rocks), mineralogical heterogeneity can result in preferential removal of the more reactive materials and lead to surface roughening. This roughening increases the contact between the fluid and the slower dissolving minerals [47]. Natural porous media usually consist of multiple mineral phases that differ in reactivity. It is thus expected that a single dissolutive percolation could display different trends representing the concentrations of dissolution products from the various minerals. For example, Noiriel et al. observed a decrease in the dissolution rate of micrite (fast) and an increase in sparite (slow) over time in the same percolation experiment [40].

Fig 6 also suggests that the effects of the global constraint, represented by *Q*, and of the system size, represented by *l*_*n*_, can be equivalent. Greater *l*_*n*_ corresponds to smaller *Q* given the same chemical free energy input, because it provides a longer residence time for the reactive fluid. A small system (*l*_*n*_ = 0.1, Fig 6C and 6D) dissolves homogeneously while a large system (*l*_*n*_ = 10) demonstrates both homogeneous and heterogeneous regimes separated by the dissolution front. Experimentally the effect of *Q* can be measured by a macroscopic Damköhler number. This number can be changed through tuning the reaction rate e.g. by manipulating the partial pressure of CO_2_ [41] or by varying residence time (by adjusting the flow rate) [39]. However, this number reflects the relative amount of the two forms of energy a system can dissipate (mechanical vs chemical) and should not be considered identical to the local Damköhler number (Fig 5E) that controls local reactant conversion.

Last but not least, we would like to point out that this study does not include a systematic investigation of *φ*’s impact. The spatial distribution of *φ* defines the initial geometry. This distribution is determined by the imaging technology and is sample specific. Our reactor network model has three conceptual components *i*) the reactant conveyer network, which distributes fluid influx among all possible pathways; *ii*) the kinetic module, which determines how fast solid depletes spatially and *iii*) the voxel level mass balancing, which defines how the results of chemical reaction are reflected in the temporal evolution of voxel porosity. The initial geometry affects the first component, i.e. the flow field. An improved imaging technology provides a more realistic field, while a different microstructure sample yields a different flow field. We have previously analysed the effect of imaging resolution in two numerical contexts. We used a conventional CFD approach (finite volume method to solve the Stokes equation on binarised tomogram [48]) to show that the influence is rock type specific and is predominately reflected in the interconnectivity of pore spaces that require discretisation. We have also applied the reactor network model on tomographic data at three different resolutions [34]. The results suggested that both the effect of the voxel size and that of the initial microstructure are secondary compared to the apparent solubility of the solid in the flowing fluid. It is thus important to ask if any variation in the initial flow field, caused by the different geometries, changes the qualitative behaviour of entropy production. I.e. is the three-stage pattern in localised rock dissolution conditioned on a very specific flow field? We argue that this is not the case. The pattern discussed above stems from the positive coupling between mineral dissolution and fluid advection, which is a result of three facts. First, within the same flow field fluid tends to flow through more porous materials because they are more permeable. Second, the rate of mineral dissolution is affected by the residence time of the fluid. Third, the presence of inherent transport heterogeneities breaks the symmetry of the geometry. A variation in the initial flow field does not negate any of these.

## Conclusions

We present an analysis of the patterns of entropy production in a dissolving natural porous medium, using a fixed initial microstructure. This approach uses a greyscale digital model of chalk obtained using X-ray nanotomography. Greyscale models allow the preservation of structural heterogeneities with very high fidelity. This is important for simulating systems in which local differences in material density can be amplified. We studied two types of entropy production: the percolative entropy generated by dissipating the kinetic energy of fluid flow and the reactive entropy that originates from the consumption of chemical free energy. By analysing these temporal patterns, we identified three distinct stages of microstructural evolution in a dissolving porous medium: induction, breakthrough and stabilisation. We found that local mixing dampens fluid channelling by reducing variations in reactant concentration. In addition, we show that the microstructural evolution can be particularly sensitive to initial transport heterogeneities when the global flow rate is small. This dependence on flow rate indicates that, to make accurate predictions, resolving microstructure is essential when the residence time of the fluid is long. We conclude the discussion by presenting a few examples of how patterns of entropy production can explain the diversified experimental observations and interpretations of water-rock interaction in Nature.
